# Comparative complications of prepectoral versus subpectoral breast reconstruction in patients with breast cancer: a meta-analysis

**DOI:** 10.3389/fonc.2024.1439293

**Published:** 2024-08-26

**Authors:** Yongxiao Wu, Lizhi Yu, Miaoyan Huang, Yanping Huang, Chunyan Li, Yiwen Liang, Weiming Liang, Tian Qin

**Affiliations:** The First Affiliated Hospital of Guangxi University of Science and Technology, Guangxi University of Science and Technology, Liuzhou, Guangxi, China

**Keywords:** breast cancer, reconstruction, prepectoral, subpectoral, complication, meta-analysis

## Abstract

**Introduction:**

This meta-analysis aims to evaluate the complications associated with prepectoral breast reconstruction (PBR) compared to subpectoral breast reconstruction (SBR) in patients diagnosed with breast cancer.

**Materials and methods:**

A comprehensive search was performed in four databases, including Medline, Embase, Web of Science and CENTRAL, to collect literature published up until December 31, 2024. In addition, we conducted a thorough manual examination of the bibliographies of the identified papers, as well as pertinent reviews and meta-analyses. We conducted a search on three clinical trial registries, namely ClinicalTrials.gov, Controlled-trials.com, and Umin.ac.jp/ctr/index.htm. Meta-analyses were conducted on total complications, hematoma, infection, wound healing issues, necrosis, capsular contracture, rippling, animation deformity, and reoperation.

**Results:**

A total of 40 studies were included in the meta-analysis. Compared with SBR, PBR significantly reduced the incidence of animated malformations (OR=0.37, 95% CI: 0.19 to 0.70, P=0.003, I ²=12%), but increased the incidence of ripples (OR=2.39, 95% CI: 1.53 to 3.72, P=0.0001, I ²=10%) and seroma (OR=1.55, 95% CI: 1.02 to 2.35, P=0.04, increasing I ²=70%).

**Conclusions:**

Our findings indicate that PBR and SBR have comparable safety profiles, with similar total complication rates. Specifically, PBR is more likely to cause rippling and seroma, whereas SBR is more prone to causing animation deformity.

**Systematic review registration:**

https://www.crd.york.ac.uk/prospero/display_record.php?ID=CRD42024565837, identifier CRD42024565837.

## 1 Introduction

Breast cancer is a prevalent malignancy among women, ranking highest in newly diagnosed cases of female cancers. The incidence of breast cancer in women increases with age, particularly post-menopause, posing a significant threat to women’s health and well-being. According to a 2021 World Health Organization survey on breast cancer incidence, approximately 2.3 million women were diagnosed with breast cancer globally in 2020, with a mortality rate of about 30%. Between 2016 and 2020, around eight million women were diagnosed with breast cancer (1). In developed regions such as North America, Europe, and Australia, breast cancer remains a common cancer among women, with high annual incidence rates. Conversely, certain Asian and African countries have lower breast cancer incidence rates.

Historically, the primary treatment for breast cancer involved the highly invasive radical mastectomy, which often resulted in significant psychological distress, including feelings of humiliation and diminished self-worth due to societal stigma. This psychological burden frequently led to low self-esteem, social withdrawal, and delays in seeking necessary follow-up care (2). Recent advancements in surgical techniques, including breast-conserving surgery, breast reconstruction post-mastectomy, and breast cancer endoscopy, have improved survival rates and reduced the psychological burden on patients (3–5).

Prosthetic breast reconstruction is a major method for breast reconstruction following breast cancer surgery (6). At present, the positions for implant placement can be divided into anterior pectoralis major and inferior pectoralis major. Prepectoral Breast Reconstruction (PBR): Involves placing the implant above the pectoralis major muscle, directly under the skin and subcutaneous tissue. PBR has gained popularity with the advent of advanced surgical techniques and improved implant technology. This method avoids disruption of the pectoralis major muscle, potentially reducing postoperative pain. PBR may lead to a quicker recovery and less postoperative discomfort, but it requires adequate soft tissue coverage and careful patient selection to minimize the risk of complications such as implant visibility and rippling. Subpectoral Breast Reconstruction (SBR): Involves placing the implant beneath the pectoralis major muscle. Traditionally, it has been the standard approach due to the muscle providing additional coverage and support for the implant. This method may reduce the risk of implant visibility and palpable edges, potentially leading to more natural aesthetic outcomes. However, SBR can be associated with postoperative pain and longer recovery times due to the manipulation of the pectoralis major muscle. This approach can be classified into immediate reconstruction, delayed reconstruction, and phased immediate reconstruction based on the timing, and into autologous tissue reconstruction, prosthesis reconstruction, and a combination of both based on the materials used (7).

Subpectoral breast reconstruction has traditionally been favored due to its provision of better vascularized soft tissue coverage. However, plastic surgeons have increasingly preferred prepectoral breast reconstruction to reduce animation deformity, perioperative narcotic use, and chest wall morbidity (2, 8). PBR was widely used in the early stages of alloplastic surgery but posed risks such as implant exposure, skin breakdown, wrinkling, rippling, palpability, visibility, and misalignment (6, 9). While the subpectoral plane offers advantages over the prepectoral plane, it also presents challenges such as pain, mobility issues, and insufficient breast projection (7, 10). The choice between SBR and PBR remains a topic of ongoing debate among surgeons. While SBR has been the traditional approach with a well-documented safety profile, PBR offers potential advantages in terms of reduced postoperative pain and quicker recovery. However, PBR’s long-term outcomes and complication rates compared to SBR are not yet fully understood, necessitating further research. By synthesizing available data, a meta-analysis can inform evidence-based clinical practices, guiding surgeons in making informed decisions about the most appropriate reconstruction technique for their patients.

## 2 Materials and methods

### 2.1 Search strategy

This meta-analysis adhered to the 2020 guidelines of the Preferred Reporting Items for Systematic Reviews and Meta-Analyses (PRISMA). The study was registered with PROSPERO (registration number CRD42024565837). We conducted a comprehensive search of PubMed, Embase, Web of Science, and the Cochrane Library for literature published up to January 31, 2024. The search strategy followed the PICOS principle, utilizing a combination of MeSH terms and free text. Keywords included “Breast Cancer”, “Mastectomy”, “Breast Implants”, “Prepectoral”, and “Subpectoral”. **Supplementary Table 1** provides a detailed search record. We also manually reviewed the bibliographies of identified studies, relevant reviews, and meta-analyses to uncover additional eligible research. Additionally, we searched three clinical trial registries: ClinicalTrials.gov, Controlled-trials.com, and Umin.ac.jp/ctr/index.htm, to include unpublished clinical studies.

### 2.2 Inclusion and exclusion criteria

The inclusion criteria are as follows: (1) Patients diagnosed with breast cancer who underwent mastectomy. (2) Intervention group patients received prepectoral prosthesis implantation. (3) Control group patients received subpectoral prosthesis implantation. (4) At least one of the following outcomes was reported: total complication, hematoma, infection, wound healing issues, necrosis, capsular contracture, rippling, animation deformity, and reoperation. (5) Study design: randomized controlled trials, prospective studies, and retrospective studies. The exclusion criteria are as follows: (1) Articles such as case reports, protocols, letters, editorials, comments, reviews, and meta-analyses. (2)Non-breast cancer studies. (3)Studies not comparing prepectoral versus subpectoral prosthesis implantation. (4) No relevant outcomes reported. (5) Duplicate patient cohorts. (6) Data could not be extracted.

### 2.3 Selection of studies

The literature selection process was executed using EndNote (Version 20; Clarivate Analytics) to eliminate duplicate entries. Two independent reviewers conducted the initial search. Redundant items were removed, and the titles and abstracts were screened for relevance. Subsequently, each study was classified as either included or excluded. Discrepancies were resolved through consensus, and if consensus was not achieved, a third reviewer acted as a mediator.

### 2.4 Data extraction

Data were extracted independently by two reviewers. The extracted data included: (1) Basic characteristics of the studies: author, nationality, year of publication. (2) Baseline characteristics of study subjects: age, sample size, tumor stage. (3) Outcome indicators: total complication, hematoma, infection, wound healing issues, necrosis, capsular contracture, rippling, animation deformity, and reoperation.

### 2.5 Quality assessment

The quality of the included studies was assessed independently by two reviewers using the Newcastle-Ottawa Scale (NOS) for retrospective studies. Discrepancies were resolved through discussion and consensus.

### 2.6 Statistical analysis

The study findings were analyzed using Review Manager 5.3 (Cochrane Collaboration, Oxford, UK). Continuous variables were compared using the weighted mean difference (WMD) with a 95% confidence interval (CI). Binary variables were compared using the relative ratio (RR) with a 95% CI. Medians and interquartile ranges of continuous data were converted to means and standard deviations. Statistical heterogeneity among studies was evaluated using Cochrane’s Q test and the I² index. Given the diversity of the included studies, the random effects model was primarily used. A p-value below 0.05 was considered statistically significant.

## 3 Results

All results of the meta-analysis were summarized in **Table 1**.

### 3.1 Search results

**Figure 1:**
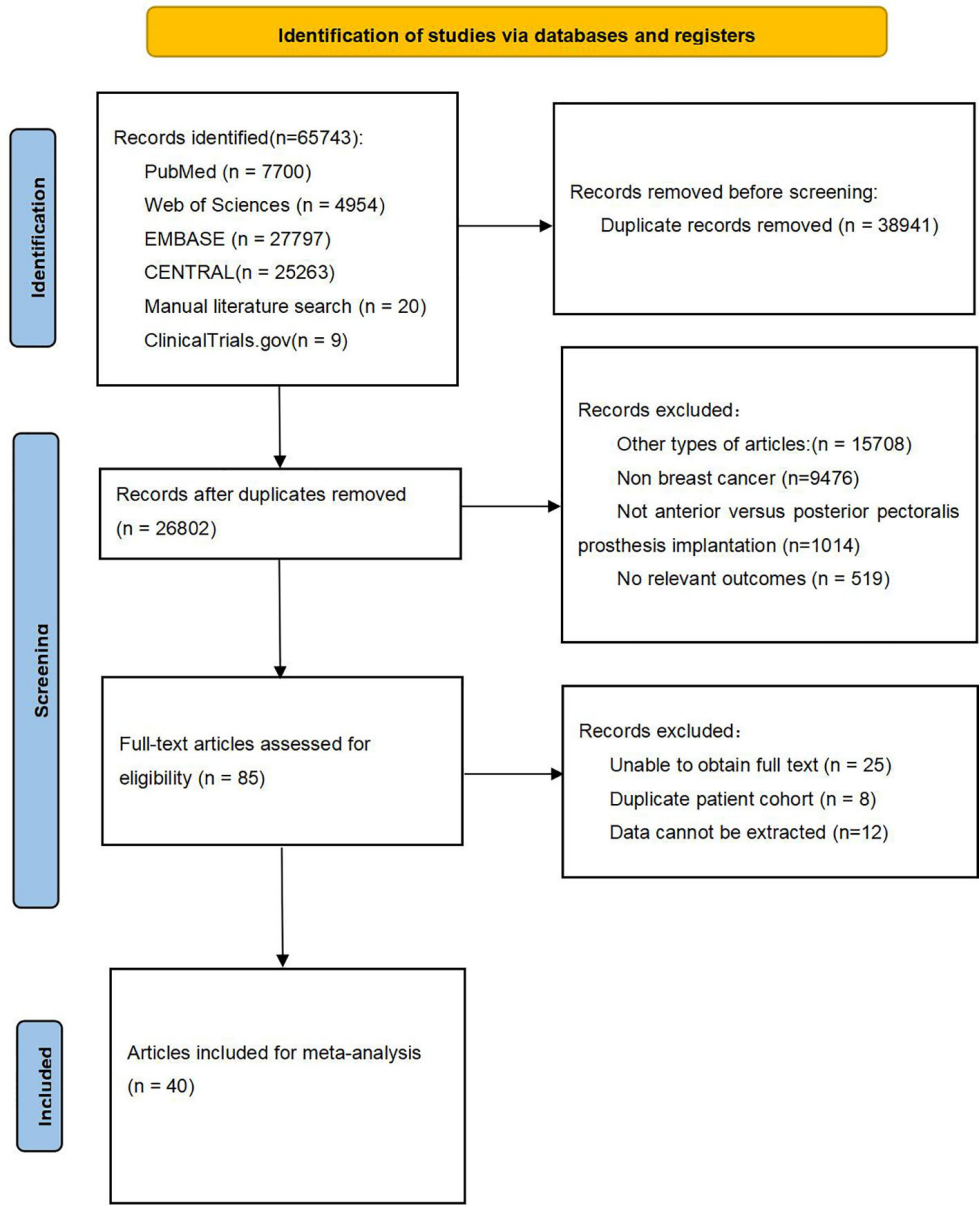
Flow chart of literature search strategies.

A total of 65,743 publications were retrieved from four databases and three clinical trial registries. After applying inclusion and exclusion criteria, 40 articles (6, 11–49) were included in the final meta-analysis. The selection and inclusion process are illustrated in **Figure 1**.

### 3.2 Study characteristics

The meta-analysis included 40 studies: 5 prospective and 35 retrospective studies, with a total of 8,632 participants. A total of 12,943 breasts underwent prosthesis implantation, with 6,749 in the PBR group and 6,194 in the SBR group. The studies were conducted in the USA, UK, France, Germany, Italy, Canada, Poland, and Korea. Detailed patient characteristics are provided in **Table 1**, with additional details in **Supplementary Table 2**.

**Table 1:** Results of the meta-analysis.

Outcomes	No. of studies	Sample size		Heterogeneity		Overall effect size	95% CI of overall effect	P Value
		PBR	SBR	I^2^(%)	P Value			
Total complication	19	1910	1749	28	0.13	OR = 1.11	0.96 ~ 1.27	0.15
Hematoma	27	3046	2857	4	0.41	OR = 0.84	0.62 ~ 1.14	0.27
Seroma	26	2593	2276	70	<0.001	OR = 1.55	1.02 ~ 2.35	0.04
Infection	30	3019	3058	13	0.26	OR = 1.03	0.85 ~ 1.26	0.75
Difficulty in wound healing	16	1857	1710	0	0.46	OR = 1.03	0.75 ~ 1.40	0.87
Ischemic necrosis	29	3057	2933	55	<0.001	OR = 0.80	0.55 ~ 1.16	0.25
Capsular contracture	17	4557	3692	74	<0.001	OR = 1.11	0.65 ~ 1.92	0.70
Rippling	7	418	557	10	<0.001	OR = 2.39	1.53 ~ 3.72	<0.001
Animation deformity	5	426	546	12	0.34	OR = 0.37	0.01 ~ 4.26	0.003
Reoperation	11	1056	1408	75	<0.001	OR = 0.94	0.58 ~ 1.53	0.80

### 3.3 Quality assessment

The quality assessment, using the NOS, rated two studies at 9 points, sixteen studies at 8 points, thirteen studies at 7 points, and nine studies at 6 points, indicating high quality for all included studies. Detailed quality assessments are presented in **Table 2**.

**Table 2:** Characteristics of included studies and patients.

Study	Country	Study design	Patients	Breast	Mean Age (years)	Mean BMI (kg/m2)	Quality (NOS)	Outcomes
Vazquez 1987 (16)	USA	R	89	100/96	31.9	NA	7	①⑥
Gruber 1981 (6)	USA	R	84	30/19	NA	NA	6	⑥
Calobrace 2018 (14)	USA	R	2565	2856/2266	36	20.8	8	⑥
Asaad 2023 (15)	USA	R	396/85	573/121	50.4/50	27/25.1	8	①②③④⑤⑥
Manrique 2020 (13)	USA	R	33/42	55/69	54/47	20.3/21	8	①③④⑤⑥
Potter 2019 (12)	UK	P	223	42/181	48/49	23.8/24.0	9	③
Yang 2019 (11)	Korea	R	79	32/47	48.9/46.4	23.49/21.25	7	①②③④⑤⑥⑦⑧
Talwar 2023	USA	R	86/87	146/146	50.2/50.4	27/27.2	7	①②③④⑤⑥
Akyurek 2019	USA	R	33/22	50/36	52.4/52.5	27.6/25.2	7	①②③⑥⑧
Manrique 2019 (46)	USA	R	100/69	187/124	35.3/34.2	25.3/26.3	7	①②③④⑤
Houvenaeghel 2022 (45)	France	R	316	98/218	NA	NA	7	①③⑤
Baker 2018 (44)	UK	P	40	20/12	47.5/48.0	26.0/23.4	8	②③⑤
Mirhaidari 2020 (43)	USA	R	62	112/112	54/48	27/26	8	①②③⑤
Plachinski 2021 (42)	USA	R	186	83/103	47.88/49.90	28.12/26.14	7	①②③④⑤⑥⑧
Bekisz 2022 (41)	USA	R	510	50/248	52.0/49.6	28.6/24.7	8	①②③④⑤
ElSherif 2023	USA	R	119/201	203/322	48.3/48.8	25.7/24.7	7	⑤
Alcon 2023 (39)	USA	R	152	38/144	46/48	NA	8	①②③⑤⑥⑦⑧
King 2021 (38)	USA	R	228	203/202	46.5/45.9	24.0/23.7	8	①⑥⑦⑧
Braun 2020 (37)	USA	R	116/44	209/79	45/46	24/24	7	②③⑤
Avila 2020 (36)	USA	R	228	203/202	46.5/45.9	24.0/23.7	6	③④⑤
Thangarajah 2019 (35)	Germany	R	63	34/29	49.9/49.3	24.7/24.4	6	③④⑤⑥
Kim 2020 (34)	Korea	R	167	53/114	47.68/46.56	23.92/22.65	6	①②③⑤⑥
Klinger 2022 (33)	Italy	R	67	13/43	52.2/48.1	21.9/20.4	6	①②③ ④⑤⑥⑦
Bettinger 2017 (32)	USA	R	110/40	165/52	50.9/51.2	≥30 36/15	9	①②③⑤
Nelson 2022 (31)	USA	R	238	119/119	53.0/50.7	26.4/27.1	7	①②③⑤
Kraenzlin 2021 (30)	USA	R	286	169/117	48.8/49.4	27.4/27.5	7	①②③④⑤
Zhu 2016 (49)	USA	R	29/59	50/108	50.48/52.69	27.77/27.54	6	②③⑤
Wormer 2019 (29)	USA	R	32/69	60/124	48.2/49.9	29.5/26.8	6	①②③⑤
Walia 2018 (28)	USA	R	135	26/109	51.4/48.6	24.3/26.1	6	①②③④⑤
Escandón 2023 (27)	USA	R	154	77/77	51.68/52.04	27.06/25.19	7	①②③④⑤⑥
Viezel-Mathieu 2020 (26)	Canada	R	39/38	60/56	46.5/50.9	NA	7	①②③⑤⑦
Chandarana 2018 (25)	UK	R	61/69	71/83	51/50	27.32/25.08	8	①②③④⑤⑥
Casella 2014 (24)	Italy	P	34/29	39/34	47/51	23/23	8	①③④⑤
Bernini 2015 (23)	Italy	P	34/29	39/34	47/51	23/23	8	①③④⑤⑥ ⑦
Wow 2020	Poland	R	170	156/76	42/46	21.49/22.21	8	②
Sobti 2020 (21)	USA	R	20/27	32/49	52.3/49.7	28.5/24.8	8	①③⑤
Darrach 2021 (20)	USA	R	133/89	133/89	48.37/48.87	27.71/27.45	6	①⑤
Le 2021 (19)	USA	R	64/37	114/68	51.0/48.5	27.1/24.5	8	①②③⑤⑥
Cogliandro 2023 (18)	Italy	R	81	29/52	51/57.2	24/23.2	8	②⑤
Joon 2021	Korea	P	34	20/14	46.2/46.8	20.93/21.28	8	②⑤⑥⑦⑧

“/” The meaning before and after is as follows: Prepectoral/Subpectoral.

Study design: P, Prospective cohort study; R, Retrospective cohort study.

Outcomes: ①Hematoma; ②Seroma; ③Infection; ④Difficulty in wound healing; ⑤Necrosis; ⑥Capsular contracture; ⑦Rippling; ⑧Animation deformity.

### 3.4 Complications

#### 3.4.1 Total complication

**Figure 2:**
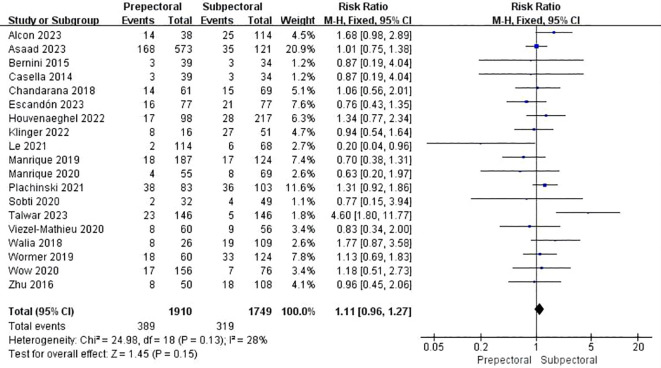
Forest plot of the meta-analysis for any complication.

Nineteen studies documented total complications (13, 15, 19, 21–29, 33, 39, 42, 45, 46, 48, 49). The pooled analysis showed no significant difference between PBR and SBR (OR = 1.11, 95% CI: 0.96 to 1.27, P=0.15, I²=28%) (**Figure 2**).

#### 3.4.2 Hematoma

**Figure 3:**
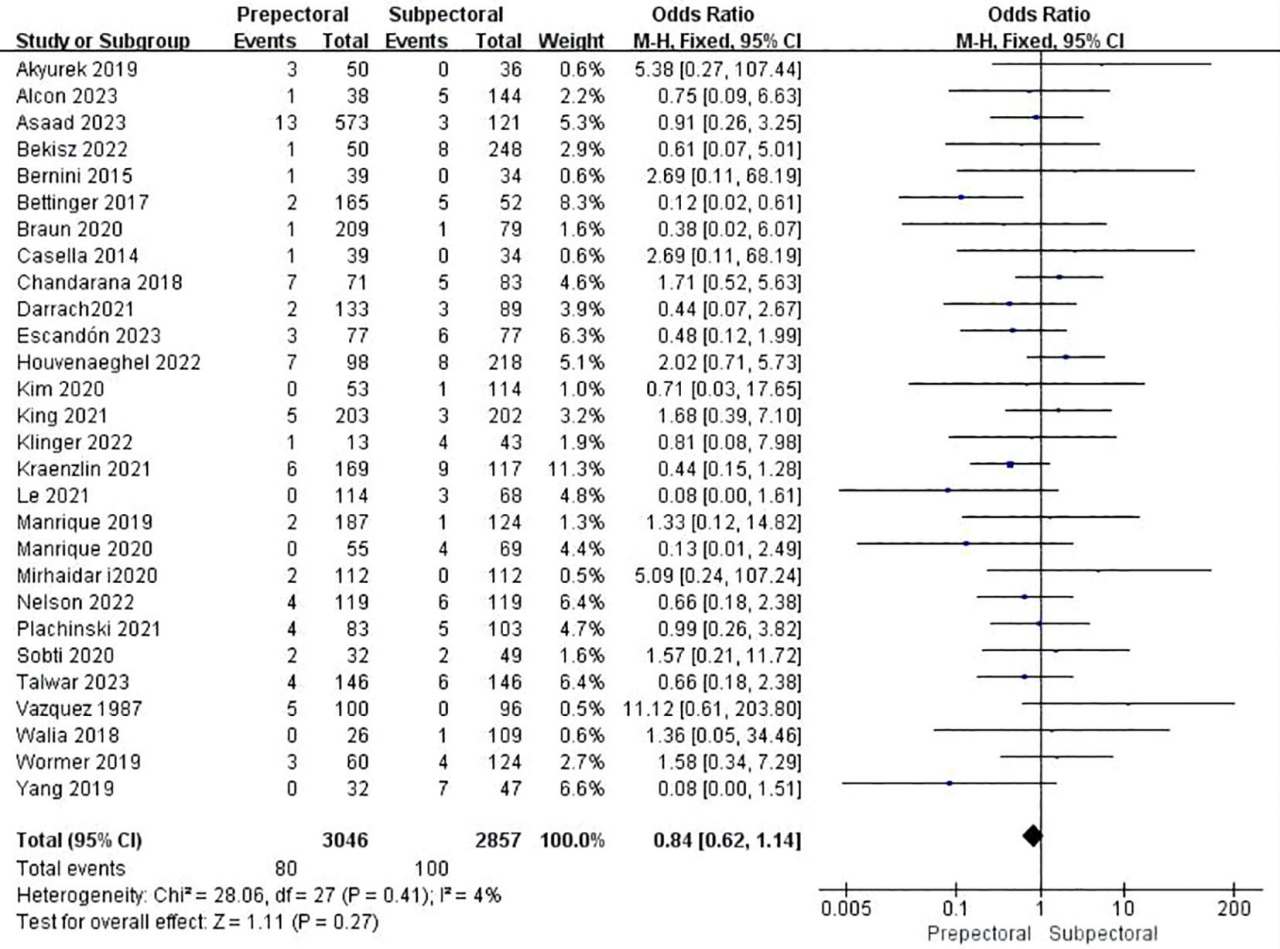
Forest plot of the meta-analysis for hematoma.

Twenty-eight studies reported hematoma (11, 13, 15, 16, 19–21, 23–34, 38, 39, 41–43, 45–48). The pooled analysis indicated no significant difference between PBR and SBR (OR = 0.84, 95% CI: 0.62 to 1.14, P=0.27, I²=4%) (**Figure 3**).

#### 3.4.3 Seroma

**Figure 4:**
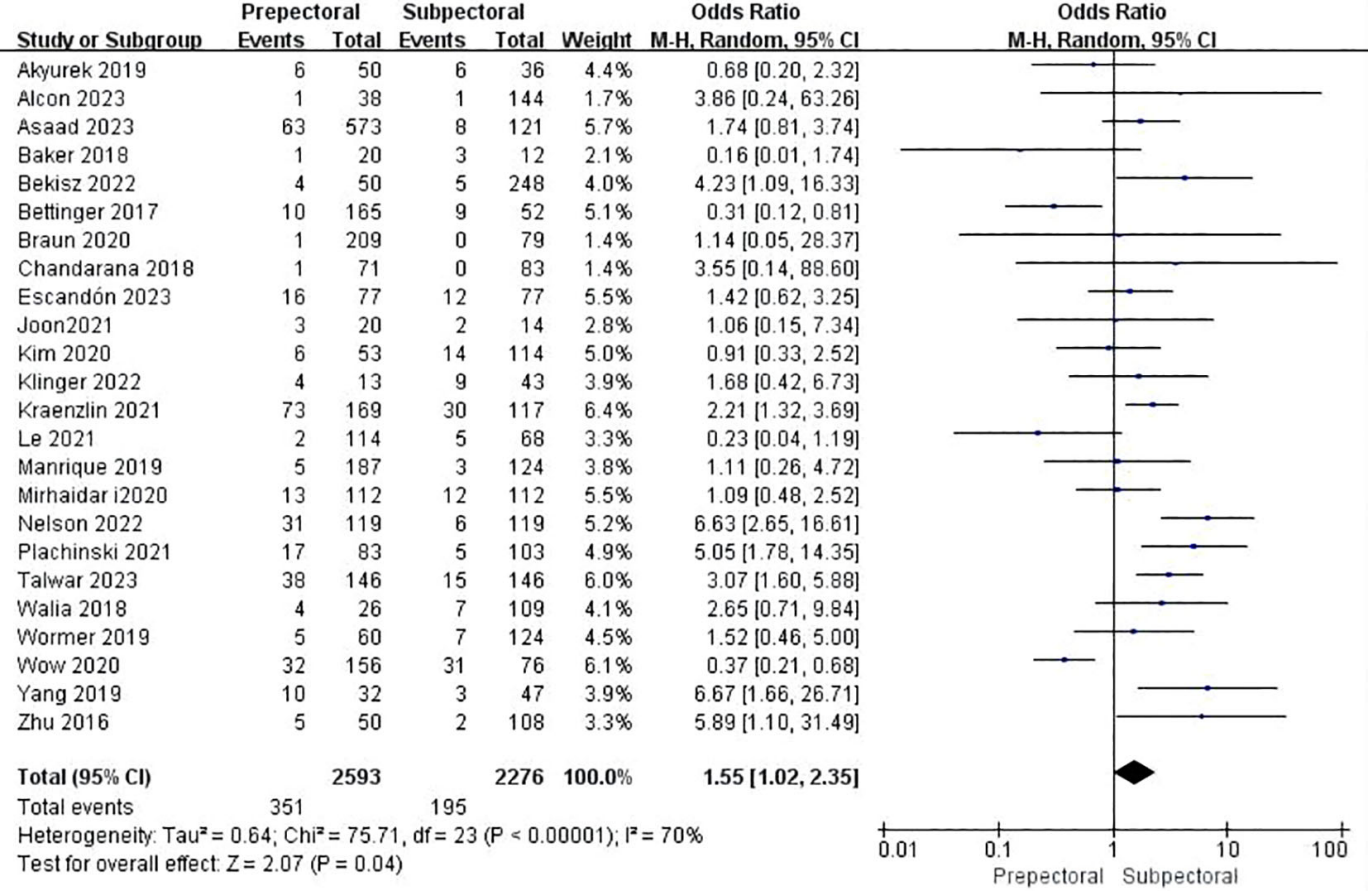
Forest plot of the meta-analysis for seroma.

Twenty-six studies reported seroma (11, 15, 17–19, 22, 25–34, 37, 39, 41–44, 46–49). The pooled analysis showed a significantly higher occurrence of seroma in the PBR group (OR = 1.55, 95% CI: 1.02 to 2.35, P=0.04, I²=70%) (**Figure 4**).

#### 3.4.4 Infection

**Figure 5:**
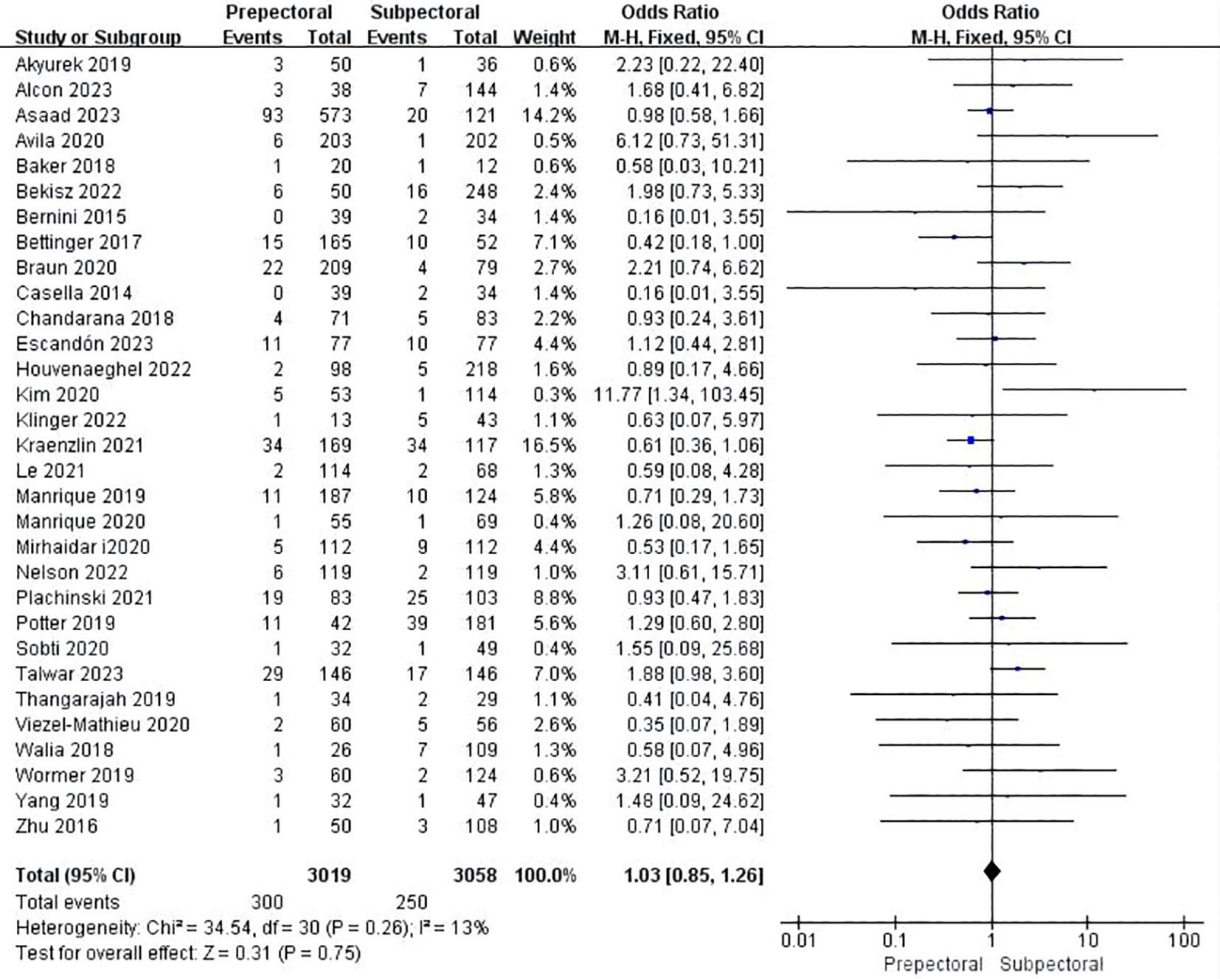
Forest plot of the meta-analysis for infection.

Thirty studies reported infection (11–13, 15, 19, 21, 23–37, 39, 41–49). The pooled analysis indicated no significant difference between PBR and SBR (OR = 1.03, 95% CI: 0.85 to 1.26, P=0.73, I²=13%) (**Figure 5**).

#### 3.4.5 Wound healing issues

**Figure 6:**
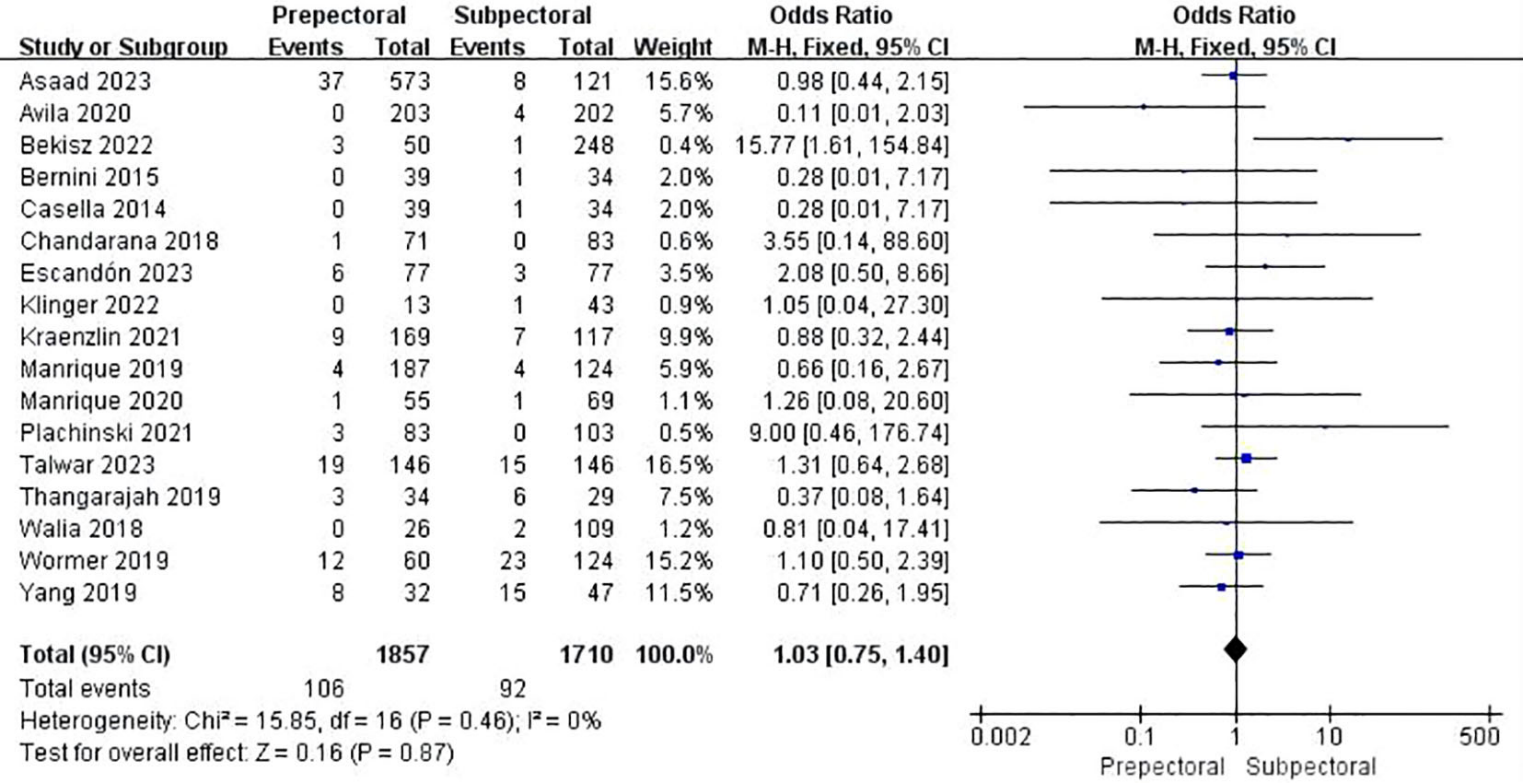
Forest plot of the meta-analysis for wound healing issues.

Sixteen studies reported wound healing issues (11, 13, 15, 23–25, 27, 28, 30, 33, 35, 36, 41, 42, 46, 48). The pooled analysis showed no significant difference between PBR and SBR (OR = 1.03, 95% CI: 0.75 to 1.40, P=0.87, I²=0%) (**Figure 6**).

#### 3.4.6 Necrosis

**Figure 7:**
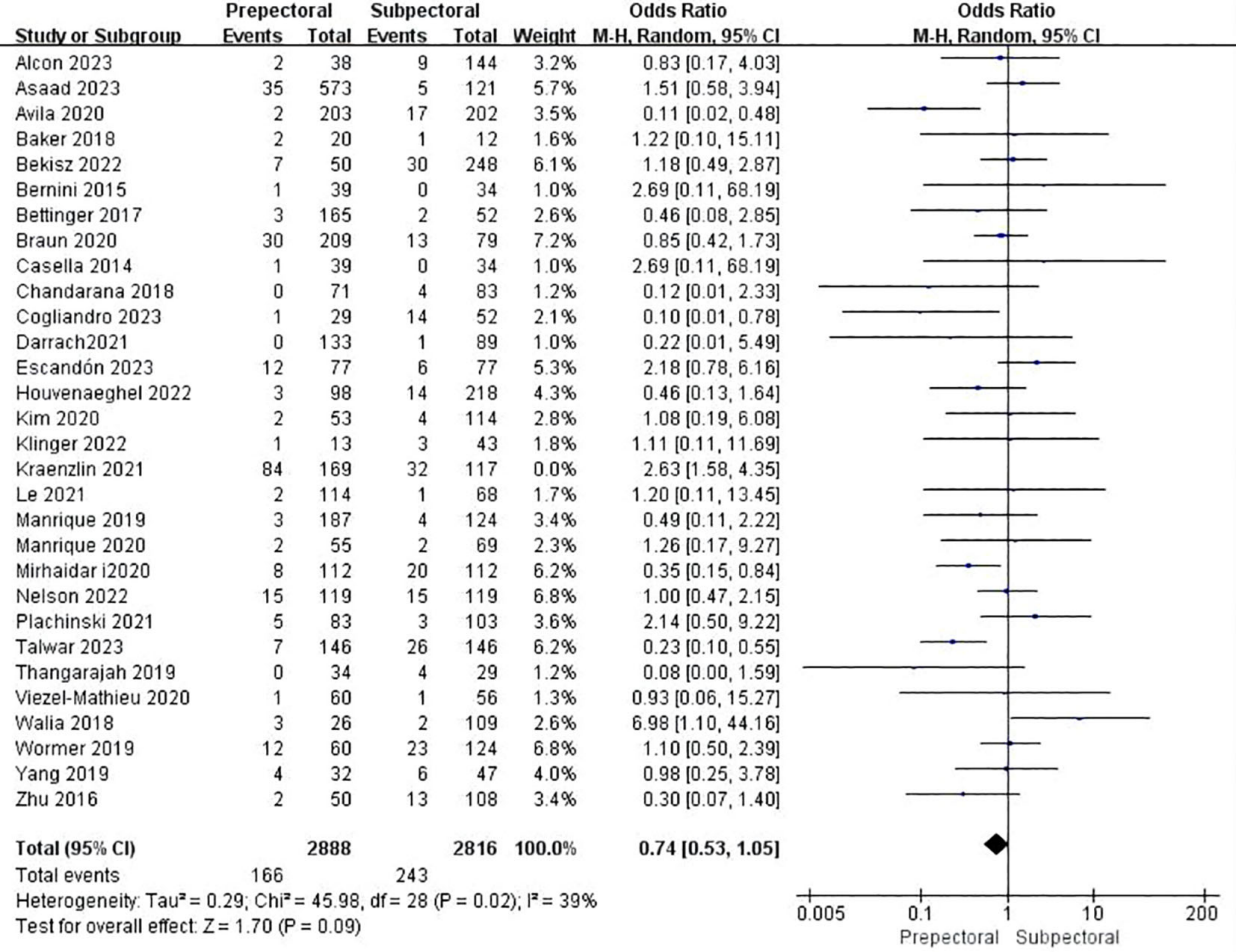
Forest plot of the meta-analysis for necrosis.

Thirty studies reported necrosis (11, 13, 15, 18–20, 23–37, 39, 41–46, 48, 49). The pooled analysis showed no significant difference between PBR and SBR (OR = 0.74, 95%CI: 0.53 to 1.05, P=0.09, I^2^ = 39%) (**Figure 7**).

#### 3.4.7 Capsular contracture

**Figure 8:**
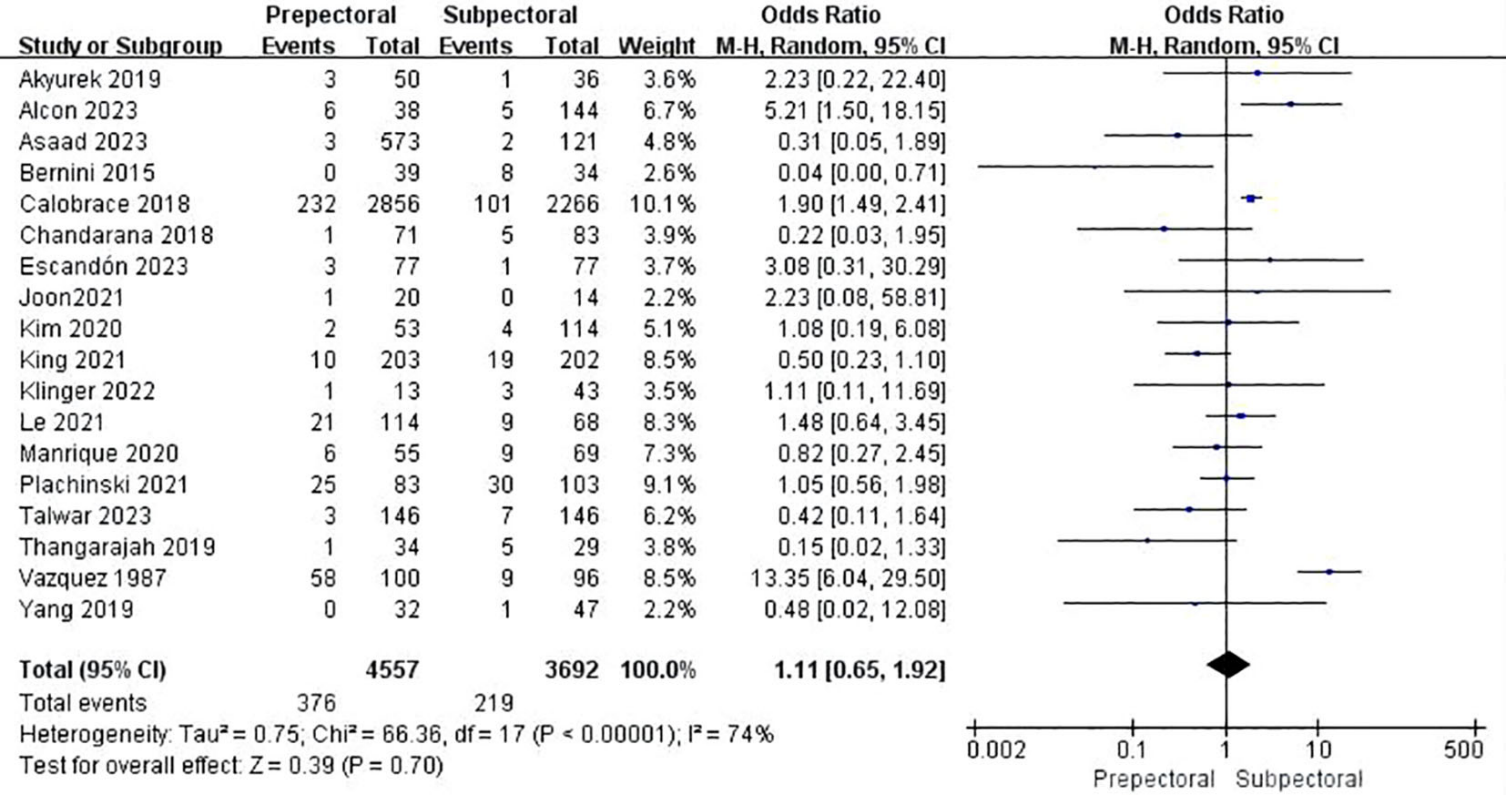
Forest plot of the meta-analysis for capsular contracture.

Eighteen studies reported capsular contracture (11, 13–17, 19, 23, 25, 27, 33–35, 38, 39, 42, 47, 48). The pooled analysis showed no significant difference between PBR and SBR (OR = 1.11, 95% CI: 0.65 to 1.92, P=0.70, I²=74%) (**Figure 8**).

#### 3.4.8 Rippling

**Figure 9:**
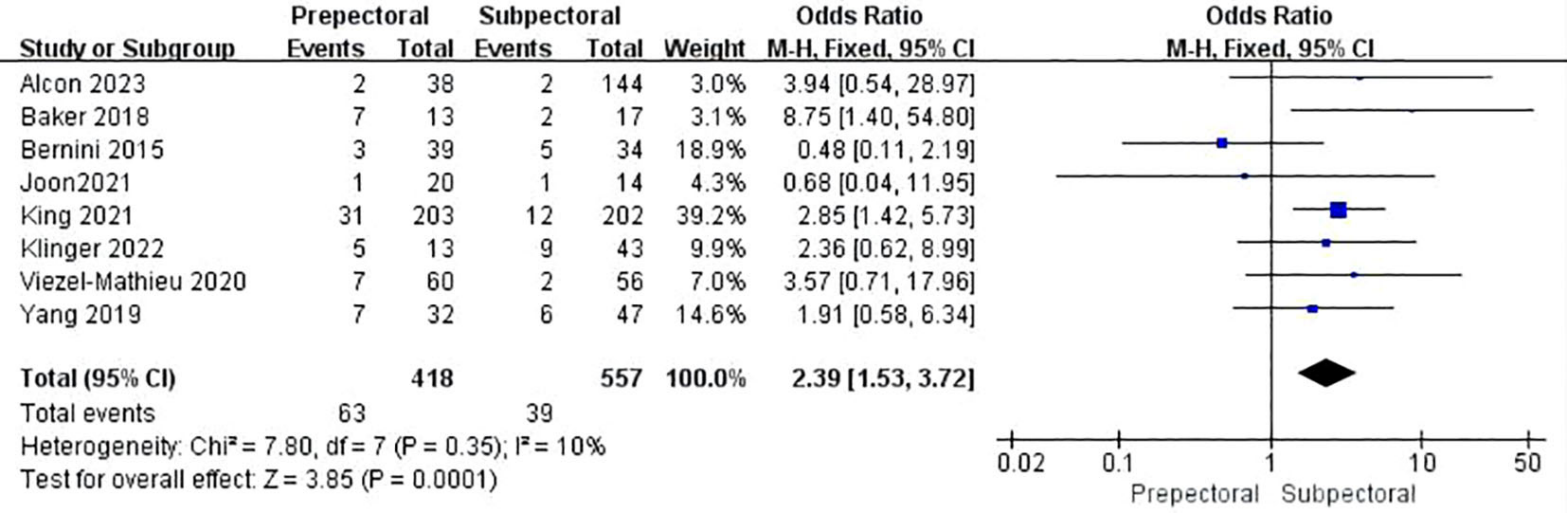
Forest plot of the meta-analysis for rippling.

Seven studies reported rippling (11, 17, 23, 26, 33, 38, 39). The pooled analysis showed a significantly higher incidence of rippling in the PBR group compared to the SBR group (OR = 2.39, 95% CI: 1.53 to 3.72, P=0.0001, I²=10%) (**Figure 9**).

#### 3.4.9 Animation deformity

**Figure 10:**
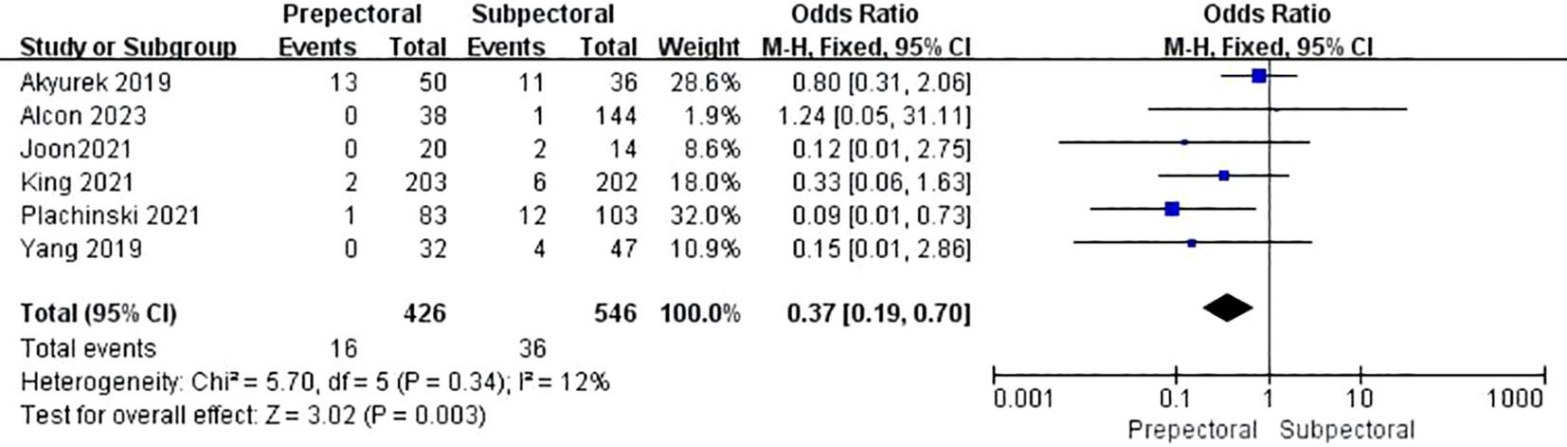
Forest plot of the meta-analysis for animation deformity.

Five studies reported animation deformity (11, 17, 38, 39, 42). The pooled analysis showed a significantly lower occurrence of animation deformity in the PBR group compared to the SBR group (OR = 0.37, 95% CI: 0.19 to 0.70, P=0.003, I²=12%) (**Figure 10**).

#### 3.4.10 Reoperation

**Figure 11:**
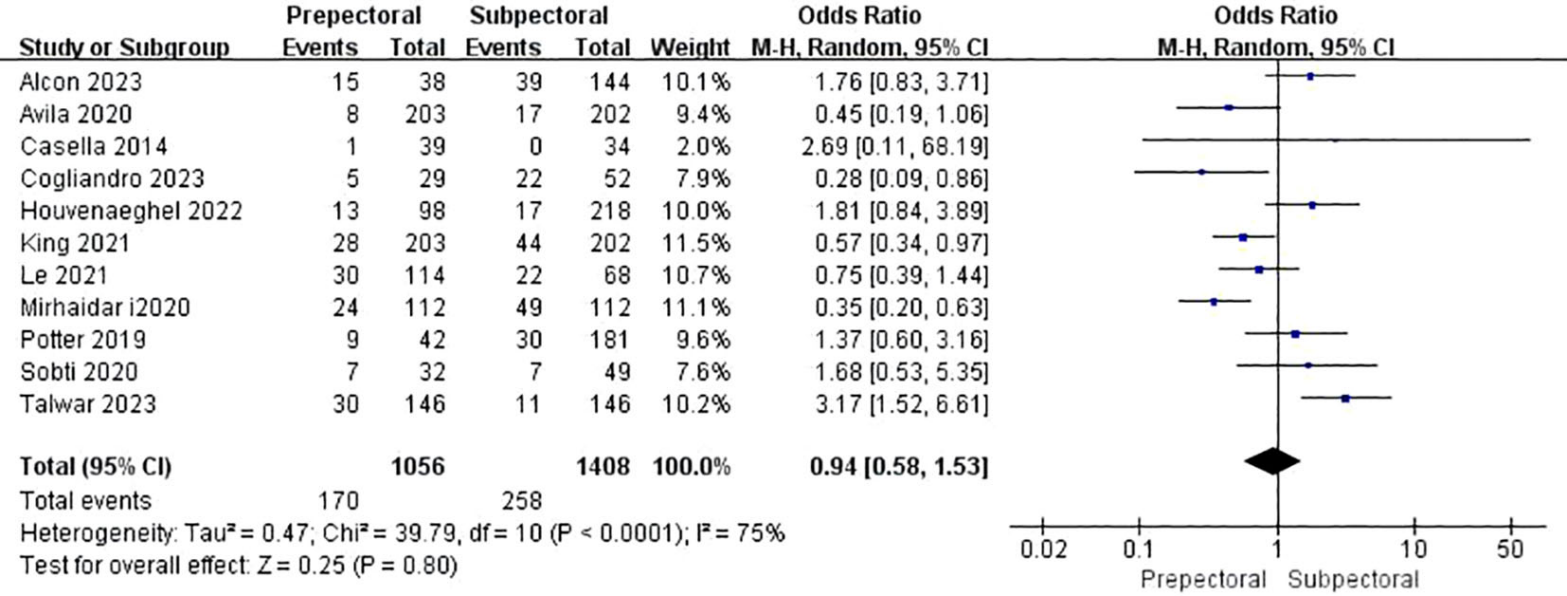
Forest plot of the meta-analysis for reoperation.

Eleven studies reported reoperation (12, 18, 19, 21, 24, 36, 38, 39, 43, 45, 48). The pooled analysis showed no significant difference between PBR and SBR (OR = 1.11, 95% CI: 0.96 to 1.27, P=0.15, I²=28%) (**Figure 11**).

### 3.5 Publication bias

**Figure 12:**
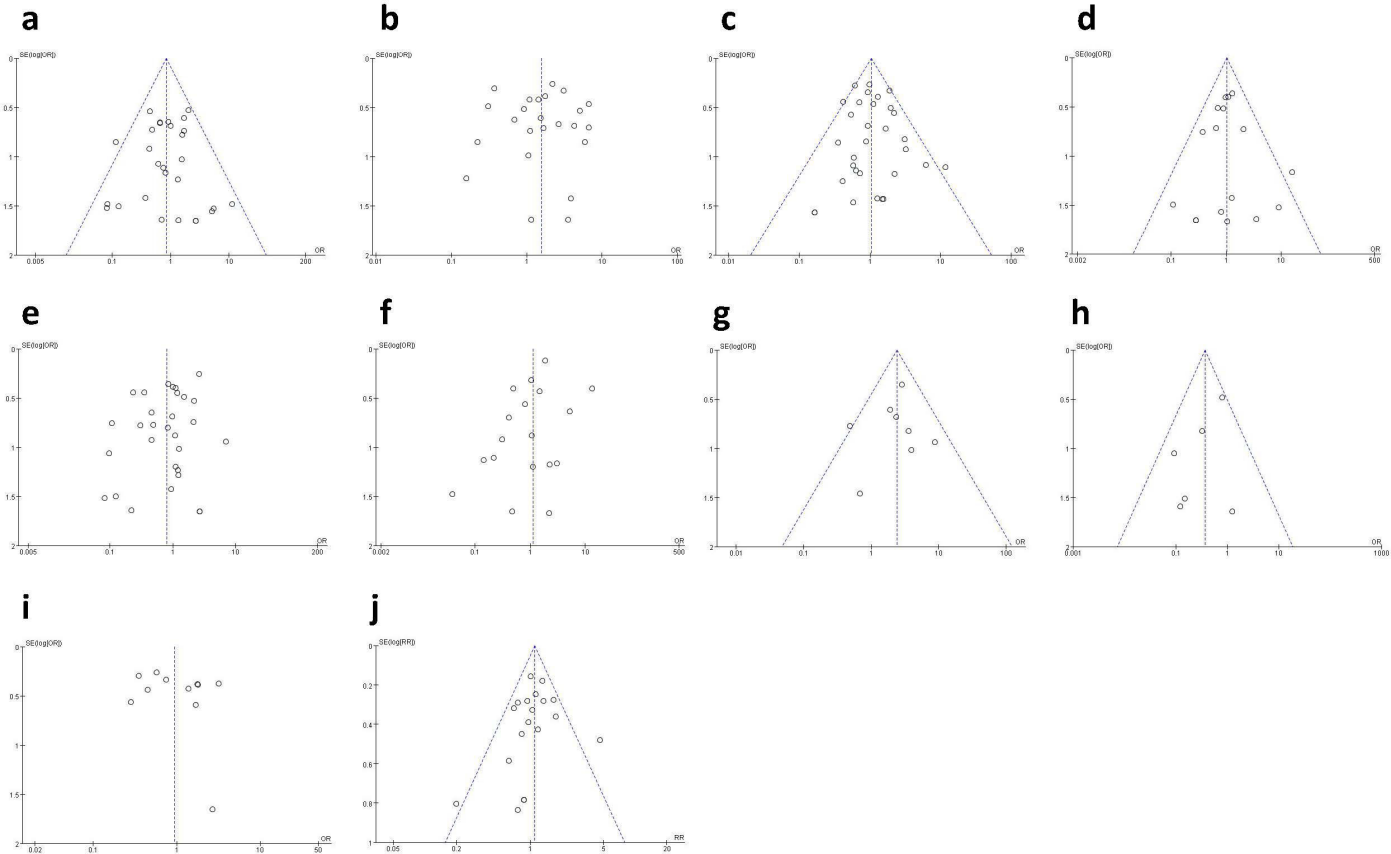
Funnel plot: **(A)** funnel plot for hematoma; **(B)** funnel plot for seroma; **(C)** funnel plot for infection; **(D)** funnel plot for wound healing issues; **(E)** funnel plot for necrosis; **(F)** funnel plot for capsular contracture; **(G)** funnel plot for rippling; **(H)** funnel plot for reoperation; **(I)** funnel plot for animation deformity; **(J)** funnel plot for total complication.

Funnel plots (**Figure 12**) were used to evaluate publication bias. The symmetrical funnel plots indicated no apparent publication bias for hematoma (**Figure 12A**), seroma (**Figure 12B**), infection (**Figure 12C**), wound healing issues (**Figure 12D**), rippling (**Figure 12G**), reoperation (**Figure 12H**), animation deformity (**Figure 12I**), and total complication (**Figure 12J**). However, the funnel plots for necrosis (**Figure 12E**) and capsular contracture (**Figure 12F**) showed significant asymmetry, indicating potential publication bias.

## 4 Discussion

This meta-analysis aimed to evaluate the complications associated with prepectoral breast reconstruction (PBR) compared to subpectoral breast reconstruction (SBR) in breast cancer patients. Our results indicated no statistically significant difference between the two groups concerning overall complications, hematoma, infection, wound healing issues, reoperation, animation abnormalities, necrosis, and capsular contracture. However, the aggregated data revealed a significantly higher incidence of seroma in the PBR group compared to the SBR group. Additionally, there was a markedly higher occurrence of rippling in the PBR group compared to the SBR group.

Implant-based breast reconstruction was initially performed from the 1960s to 1970s, predominantly utilizing the pectoralis major muscle. The first documentation of this procedure appeared in 1971. However, it was discontinued due to the emergence of numerous complications (2, 50). To prevent ripple deformity and the development of capsular contracture, a subpectoral major implant graft was devised (6, 51) Direct subpectoral muscle reconstruction is becoming increasingly common in many medical institutions. This procedure involves placing a permanent implant or expander along the breast using a biomaterial or synthetic mesh, typically following breast cancer treatment (52). Subpectoral major muscle implantation can effectively address several complications. However, it may also lead to animation deformity or breast deformity due to the contraction of the chest muscle after subpectoral muscle reconstruction surgery. This, in turn, can result in new issues, primarily recurrent pain (10, 53–55).

Due to advancements in technology for pectoralis major anterior implant grafts, many surgeons have reevaluated the positioning of implants in the chest plane (56). Additionally, the integration of contemporary tissue vascularization techniques and the utilization of novel surgical materials have been combined to enhance the outcomes of prepectoral restoration (57). Hence, the selection of the implantation plane should be approached with careful consideration, as each plane offers distinct advantages and noticeable drawbacks. Wrapping acellular dermal matrix (ADM) improves the resolution of issues. The use of ADM has been found to significantly decrease the incidence of capsular contracture, potentially due to a reduction in the production of granulation tissue (21, 58, 59). ADM can be derived from human, bovine, or porcine sources and must undergo biotechnological processes to eliminate cell antigens and prevent antibody reactions. However, it retains a structural matrix that supports and enhances tissue regeneration (25). Despite these advancements, patients undergoing breast reconstruction still face substantial issues such as animation deformity, corrugated malformations, and capsular contracture. The advancement of the times has facilitated progress in tissue expanders, ADM, and breast cancer surgery, thus propelling the advancement of implant-based reconstructive surgery (13, 60, 61).

Previous meta-analyses have compared prepectoral breast reconstruction (PBR) with subpectoral breast reconstruction (SBR). A meta-analysis by Li et al. found no significant differences in overall complications between PBR and SBR. Additionally, there were no significant differences in the incidence of tissue necrosis, hematoma, seroma, infection, and wound dehiscence. However, the incidence of capsular contracture was lower in the PBR group compared to the SBR group (62). The meta-analysis also found that capsular contracture (OR 0.26) and hematoma (OR 0.35) were significantly lower in SBR compared with PBR, but there was a higher incidence of implant displacement (OR) and animation deformity (OR 14.47). No significant differences were found in seroma (OR 1.06), ripple deformity (OR 1.39), and infection (OR 1.21) between the two groups. Implant-based breast reconstruction carries a risk of tissue ischemia and necrosis, which is increased by conditions such as patient smoking, advanced age, hypertension, diabetes, and obesity (63, 64). Additionally, several studies have documented that the surgical approach plays a crucial role in tissue necrosis (65, 66). The surgical approach is a crucial determinant of tissue necrosis. For instance, the selection of the incision type and the decrease in the thickness of the mastectomy flap are factors that influence the eventual loss of blood supply to the tissue, resulting in local ischemia and tissue necrosis (65, 66). Another meta-analysis (67) revealed that the incidence of capsular contracture (OR 0.26) and hematoma (OR 0.35) was significantly reduced in SBR compared to PBR. The results of capsule contracture are vastly different from previous clinical and evidence-based medicine conclusions. However, there was a higher likelihood of implant displacement (OR) and animation deformity (OR 14.47) in SBR. No significant differences were found in the incidence of seroma (OR 1.06), ripple deformity (OR 1.39), and infection (OR 1.21) between the two groups. Chatterjee et al. (68) conducted a meta-analysis of 14 trials with a total of 654 breasts. The study revealed that tissue necrosis was the most prevalent problem prior to chest reconstruction, occurring in 7.8% of cases. Seroma and capsular contracture followed closely behind, with incidences of 6.7% and 5.8% respectively. No notable disparities were observed in the rates of infection (OR 0.46) and dehiscence (OR 1.84). It is important to mention that our study incorporated a significantly larger number of articles compared to previous meta-analyses. This enables us to obtain more dependable and trustworthy conclusions.

The SBR approach is widely recognized as a significant risk factor for animation deformity. This is due to its impact on the stability of the pectoralis major muscle in its natural state, leading to the repair and fibrosis of the muscle (69). Consequently, this results in an unnatural change in breast shape when the muscle contracts. Animation deformity negatively impacts aesthetics and significantly impairs quality of life and comfort. Becker et al. found that 80% of surveyed patients experienced negative effects due to animation deformity, with 45% experiencing substantial impacts (70). Furthermore, nearly half of the patients reported that animation deformity negatively impacted their academic and professional pursuits, as well as their daily activities. Additionally, approximately one-third of patients expressed a desire to undergo reconstructive treatment when surveyed.

This meta-analysis includes the highest number of studies comparing the complications of PBR and SBR in the treatment of breast cancer, to the best of our knowledge. This has the potential to result in more dependable conclusions. Our findings provide valuable insights into the clinical outcomes of surgical procedures that contribute to both clinical practice and research in the field of breast cancer. However, we acknowledge the possible limitations of our research. Firstly, the studies included in the analysis were not randomized controlled trials (RCTs), but rather prospective or retrospective investigations. This introduces the possibility of selective bias in patient selection, which reduces the reliability and trustworthiness of the findings. Furthermore, the omission of confounding factors, such as differences in nations, case inclusion criteria, medical equipment, adjuvant therapy, mastectomy, implant, mean implant size, and surgical procedures, can result in research heterogeneity and bias.

In conclusion, our research indicates that both PBR and SBR have similar safety profiles, with comparable incidence rates of total complications. More precisely, PBR is more susceptible to rippling and seroma, while SBR is more susceptible to causing animation deformity.

## Data Availability

The datasets presented in this study can be found in online repositories. The names of the repository/repositories and accession number(s) can be found in the article/**Supplementary Material**.
