# Supplementary material for: Comparative complications of prepectoral versus subpectoral breast reconstruction in patients with breast cancer: a meta-analysis
Source: Front Oncol. 2024 Aug 26;14:1439293. doi: 10.3389/fonc.2024.1439293 (PMC11385603; doi:10.3389/fonc.2024.1439293)
Supplement: Supplementary file 1 [file Table1.docx]

**Supplement table 1**

| pubmed | |
| --- | --- |
| No. | Query |
| 1 | ("Breast Neoplasms"[Mesh]) OR (((((((((((((((((((((((((((((((((((((Breast Neoplasm) OR (Neoplasm, Breast)) OR (Breast Tumors)) OR (Breast Tumor)) OR (Tumor, Breast)) OR (Tumors, Breast)) OR (Neoplasms, Breast)) OR (Breast Cancer)) OR (Cancer, Breast)) OR (Mammary Cancer)) OR (Cancer, Mammary)) OR (Cancers, Mammary)) OR (Mammary Cancers)) OR (Malignant Neoplasm of Breast)) OR (Breast Malignant Neoplasm)) OR (Breast Malignant Neoplasms)) OR (Malignant Tumor of Breast)) OR (Breast Malignant Tumor)) OR (Breast Malignant Tumors)) OR (Cancer of Breast)) OR (Cancer of the Breast)) OR (Mammary Carcinoma, Human)) OR (Carcinoma, Human Mammary)) OR (Carcinomas, Human Mammary)) OR (Human Mammary Carcinomas)) OR (Mammary Carcinomas, Human)) OR (Human Mammary Carcinoma)) OR (Mammary Neoplasms, Human)) OR (Human Mammary Neoplasm)) OR (Human Mammary Neoplasms)) OR (Neoplasm, Human Mammary)) OR (Neoplasms, Human Mammary)) OR (Mammary Neoplasm, Human)) OR (Breast Carcinoma)) OR (Breast Carcinomas)) OR (Carcinoma, Breast)) OR (Carcinomas, Breast)) |
| 2 | ((("Mastectomy"[Mesh]) OR (Mastectomies)) OR (Mammectomy)) OR (Mammectomies) |
| 3 | #1 OR #2 |
| 4 | ((((((((("Breast Implants"[Mesh]) OR (Breast Prosthesis, Internal)) OR (Breast Prostheses, Internal)) OR (Internal Breast Prostheses)) OR (Internal Breast Prosthesis)) OR (Prostheses, Internal Breast)) OR (Prosthesis, Internal Breast)) OR (Implant*, Breast)) OR (Breast Implant*)) OR (Implant*, Breast) |
| 5 | ((((((((((((((((("Prostheses and Implants"[Mesh]) OR (Implants and Prostheses)) OR (Prosthetic Implants)) OR (Prostheses and Implant)) OR (Implant and Prostheses)) OR (Prosthetic Implant)) OR (Implant, Prosthetic)) OR (Implants, Prosthetic)) OR (Endoprosthesis)) OR (Endoprostheses)) OR (Prosthes*)) OR (Implants, Artificial)) OR (Artificial Implant)) OR (Artificial Implants)) OR (Implant, Artificial)) OR (Implantation, Prosthesis)) OR (Implantations, Prosthesis)) OR (Prosthesis Implantations) |
| 6 | ((((((("Mammaplasty"[Mesh]) OR (Mammaplasties)) OR (Mammoplasty)) OR (Mammoplasties)) OR (Breast Reconstruction)) OR (Breast Reconstructions)) OR (Reconstruction, Breast)) OR (Reconstructions, Breast) |
| 7 | #4 OR #5 OR #6 |
| 8 | (Prepectora) OR (Subpectoral) |
| 9 | #3 AND #7 AND #8 |

| Cochrane library | |
| --- | --- |
| No. | Query |
| 1 | MeSH descriptor: [Breast Neoplasms] explode all trees |
| 2 | (Neoplasm, Breast):ti,ab,kw OR (Breast Tumors):ti,ab,kw OR (Breast Tumor):ti,ab,kw OR (Tumor, Breast):ti,ab,kw OR (Tumors, Breast):ti,ab,kw (Word variations have been searched) |
| 3 | (Neoplasms, Breast):ti,ab,kw OR (Breast Cancer):ti,ab,kw OR (Cancer, Breast):ti,ab,kw OR (Mammary Cancer):ti,ab,kw OR (Cancer, Mammary):ti,ab,kw (Word variations have been searched) |
| 4 | (Cancer, Mammary):ti,ab,kw OR (Mammary Cancers):ti,ab,kw OR (Malignant Neoplasm of Breast):ti,ab,kw OR (Breast Malignant Neoplasm):ti,ab,kw OR (Malignant Tumor of Breast):ti,ab,kw (Word variations have been searched) |
| 5 | (Cancer of the Breast):ti,ab,kw OR (Cancer of Breast):ti,ab,kw OR (Mammary Carcinoma, Human):ti,ab,kw OR (Carcinoma, Human Mammary):ti,ab,kw OR (Human Mammary Carcinomas):ti,ab,kw (Word variations have been searched) |
| 6 | (Mammary Carcinomas, Human):ti,ab,kw OR (Human Mammary Carcinoma):ti,ab,kw OR (Mammary Neoplasms, Human):ti,ab,kw OR (Human Mammary Neoplasm):ti,ab,kw OR (Neoplasm, Human Mammary):ti,ab,kw (Word variations have been searched) |
| 7 | (Mammary Neoplasm, Human):ti,ab,kw OR (Breast Carcinoma):ti,ab,kw OR (Carcinoma, Breast):ti,ab,kw AND (Breast Carcinomas):ti,ab,kw AND (Carcinomas, Breast):ti,ab,kw (Word variations have been searched) |
| 8 | #1 or #2 or #3 or #4 or #5 or #6 or #7 |
| 9 | MeSH descriptor: [Mastectomy] explode all trees |
| 10 | (Mastectomies):ti,ab,kw OR (Mammectomy):ti,ab,kw OR (Mammectomies):ti,ab,kw (Word variations have been searched) |
| 11 | #9 or #10 |
| 12 | #8 and #11 |
| 13 | MeSH descriptor: [Breast Implants] explode all trees |
| 14 | (Breast Prosthesis, Internal):ti,ab,kw OR (Breast Prostheses, Internal):ti,ab,kw OR (Internal Breast Prostheses):ti,ab,kw OR (Internal Breast Prosthesis):ti,ab,kw OR (Prostheses, Internal Breast):ti,ab,kw (Word variations have been searched) |
| 15 | (Prosthesis, Internal Breast):ti,ab,kw OR (Implants, Breast):ti,ab,kw OR (Breast Implant):ti,ab,kw OR (Implant, Breast):ti,ab,kw OR (Prostheses and Implants):ti,ab,kw (Word variations have been searched) |
| 16 | (Implants and Prostheses):ti,ab,kw OR (Prosthetic Implants):ti,ab,kw OR (Prostheses and Implant):ti,ab,kw OR (Implant and Prostheses):ti,ab,kw OR (Prosthetic Implant):ti,ab,kw (Word variations have been searched) |
| 17 | (Implant, Prosthetic):ti,ab,kw OR (Implants, Prosthetic):ti,ab,kw OR (Endoprosthesis):ti,ab,kw OR (Endoprostheses):ti,ab,kw OR (Prostheses):ti,ab,kw (Word variations have been searched) |
| 18 | (Prosthesis):ti,ab,kw OR (Implants, Artificial):ti,ab,kw OR (Artificial Implant):ti,ab,kw OR (Artificial Implants):ti,ab,kw OR (Implant, Artificial):ti,ab,kw (Word variations have been searched) |
| 19 | #13 or #14 or #15 or #16 or #17 or #18 |
| 20 | (Prepectoral):ti,ab,kw OR (Subpectoral):ti,ab,kw (Word variations have been searched) |
| 21 | MeSH descriptor: [Mammaplasty] explode all trees |
| 22 | (Mammaplasties):ti,ab,kw OR (Mammoplasty):ti,ab,kw OR (Mammoplasties):ti,ab,kw OR (Breast Reconstruction):ti,ab,kw OR (Reconstruction, Breast):ti,ab,kw (Word variations have been searched) |
| 23 | #21 or #22 |
| 24 | #19 or #20 or #23 |
| 25 | ("complication"):ti,ab,kw (Word variations have been searched) |
| 26 | #12 and #24 and #25 |

| Embase | |
| --- | --- |
| No. | Query |
| 1 | 'breast tumor'/exp OR 'breast tumor' OR 'breast neoplasm':ti,ab OR 'neoplasm, breast':ti,ab OR 'breast tumors':ti,ab OR 'breast tumor':ti,ab 'tumor, breast':ti,ab OR 'tumors, breast':ti,ab OR 'neoplasms, breast':ti,ab OR 'breast cancer':ti,ab OR 'cancer, breast':ti,ab OR 'mammary cancer':ti,ab OR 'cancer, mammary':ti,ab OR 'cancers, mammary':ti,ab OR 'mammary cancers':ti,ab OR 'malignant neoplasm of breast':ti,ab OR 'breast malignant neoplasm':ti,ab OR 'breast malignant neoplasms':ti,ab OR 'malignant tumor of breast':ti,ab OR 'breast malignant tumor':ti,ab OR 'breast malignant tumors':ti,ab OR 'cancer of breast':ti,ab OR 'cancer of the breast':ti,ab OR 'mammary carcinoma, human':ti,ab OR 'carcinoma, human mammary':ti,ab OR 'carcinomas, human mammary':ti,ab OR 'human mammary carcinomas':ti,ab OR 'mammary carcinomas, human':ti,ab OR 'human mammary carcinoma':ti,ab OR 'mammary neoplasms, human':ti,ab OR 'human mammary neoplasm':ti,ab OR 'human mammary neoplasms':ti,ab OR 'neoplasm, human mammary':ti,ab OR 'neoplasms, human mammary':ti,ab OR 'mammary neoplasm, human':ti,ab OR 'breast carcinoma':ti,ab OR 'breast carcinomas':ti,ab OR 'carcinoma, breast':ti,ab OR 'carcinomas, breast':ti,ab |
| 2 | 'mastectomy'/exp OR 'mastectomies':ti,ab OR 'mammectomy':ti,ab OR 'mammectomies':ti,ab |
| 3 | #1 OR #2 |
| 4 | 'breast endoprosthesis'/exp OR 'breast prosthesis, internal':ti,ab OR 'breast prostheses, internal':ti,ab OR 'internal breast prostheses':ti,ab OR 'internal breast prosthesis':ti,ab OR 'prostheses, internal breast':ti,ab OR 'prosthesis, internal breast':ti,ab OR 'implants, breast':ti,ab OR 'implant, breast':ti,ab OR 'prostheses and implants':ti,ab OR 'implants and prostheses':ti,ab OR 'prosthetic implants':ti,ab OR 'prostheses and implant':ti,ab OR 'implant and prostheses':ti,ab OR 'prosthetic implant':ti,ab OR 'implant, prosthetic':ti,ab OR 'implants, prosthetic':ti,ab OR 'endoprosthesis':ti,ab OR 'endoprostheses':ti,ab OR 'prostheses':ti,ab OR 'prosthesis':ti,ab OR 'implants, artificial':ti,ab OR 'implants, artificial':ti,ab OR 'artificial implants':ti,ab OR 'artificial implants':ti,ab OR 'implantation, prosthesis':ti,ab OR 'implantations, prosthesis':ti,ab OR 'prosthesis implantations':ti,ab |
| 5 | prepectoral OR subpectoral |
| 6 | 'breast reconstruction'/exp OR 'mammaplasty':ti,ab OR 'mammaplasties':ti,ab OR 'mammoplasty':ti,ab OR 'mammoplasties':ti,ab OR 'breast reconstruction':ti,ab OR 'breast reconstructions':ti,ab OR 'reconstruction, breast':ti,ab OR 'reconstructions, breast':ti,ab |
| 7 | 'complication'/exp |
| 8 | #4 OR #5 OR #6 |
| 9 | #3 AND #8 |

| Web of science | |
| --- | --- |
| No. | Query |
| 1 | TS=(Breast Neoplasm OR Neoplasm, Breast OR Breast Tumors OR Breast Tumor OR Tumor, Breast OR Tumors, Breast OR Neoplasms, Breast OR Breast Cancer OR Cancer, Breast OR Mammary Cancer OR Cancer, Mammary OR Mammary Cancers OR Malignant Neoplasm of Breast OR Breast Malignant Neoplasm OR Malignant Tumor of Breast OR Breast Malignant Tumor OR Cancer of Breast OR Mammary Carcinoma, Human OR Carcinoma, Human Mammary OR Mammary Carcinomas, Human OR Human Mammary Carcinoma OR Mammary Neoplasms, Human OR Human Mammary Neoplasm OR Neoplasm, Human Mammary OR Mammary Neoplasm, Human OR Breast Carcinoma OR Carcinoma, Breast) |
| 2 | TS=(Mastectomy OR Mastectomies OR Mammectomy OR Mammectomies) |
| 3 | TS=(Breast Implants OR Breast Prosthesis, Internal OR Breast Prostheses, Internal OR Internal Breast Prostheses OR Internal Breast Prosthesis OR Prostheses, Internal Breast OR Prosthesis, Internal Breast OR Implants, Breast OR Breast Implant OR Implant, Breast OR Prostheses and Implant OR Implant and Prostheses OR Prosthetic Implant OR Prosthetic Implant OR Implant, Prosthetic OR Endoprosthesis OR Endoprostheses OR Prostheses OR Prosthesis OR Implant, Artificial OR Artificial Implant) |
| 4 | TS=(Prepectoral OR Subpectoral) |
| 5 | TS=(Mammaplasty OR Mammaplasties OR Mammoplasty OR Mammoplasties OR Breast Reconstruction OR Reconstruction, Breast) |
| 6 | TS=(Complication) |
| 7 | #1 AND #2 |
| 8 | #3 OR #4 OR #5 |
| 9 | #7 AND #8 AND #6 |
