# Supplementary material for: Comparative complications of prepectoral versus subpectoral breast reconstruction in patients with breast cancer: a meta-analysis
Source: Front Oncol. 2024 Aug 26;14:1439293. doi: 10.3389/fonc.2024.1439293 (PMC11385603; doi:10.3389/fonc.2024.1439293)
Supplement: Supplementary file 2 [file Table2.docx]

| **Study** | **Patients** | **Length of research** | **Follow -up** | **Diabetes** | **Hypertension** | **Tobacco use** | **Adjuvant treatment** | **Mastectomy** | **Stage** | **Implant** | **Mean**  **Implant size** |
| --- | --- | --- | --- | --- | --- | --- | --- | --- | --- | --- | --- |
| Vazquez 1987 | 89 | Between June 1980 and July 1985 | 38.1 / 17.4 months | - | - | - | - | - | - | Implants  (silicone or Saline) | - |
| Gruber 1981 | 84 | - | 1 - 5 years | - | - | - | - | - | Delay  + Single stage | Implants  (silicone, round, oval ) | - |
| Calobrace 2018 | 2565 | 10 years | at 1-year intervals (or more often, as needed) | - | - | - | - | - | Single stage | Implants  (Silicone, Smooth or Textured)  + Surgical bra | ≤355cc: 2683  ＞355cc: 2439 |
| Asaad 2023 | 396 / 85 | Between January 1, 2018, through December 31, 2019 | 15.6 / 17.9 months | 28 / 2 | 84 / 16 | 14 / 3 | Neo R: 25 / 4  R: 80 / 32  Neo C: 133 / 27  C: 106 / 23  E: 156 / 38 | SSM: 462 / 87  NSM: 107 / 33  simple: 3 / 1  Other: 1 / 0 | Immediate or Delay  + Single or Two stage | TE + ADM | - |
| Manrique 2020 | 33 / 42 | Between January of 2011 and February of 2018 | 20.3/21 months | 1 / 1 | 7 / 8 | 9 / 12 | R: 3 / 10  Neo C: 5 / 21  E: 29 / 30 | NSM: 32 / 33  SSM: 23 / 36 | Immediate  + Single stage | Implants  (silicone or Saline, Smooth or Textured, Round or Anatomical) +ADM | 410 / 425 CC |
| Potter 2019 | 223 | Between Feb 1, 2014, and June 30, 2016 | at 30 days, 3 months and 18 months after surgery | 0 / 4 | - | 11 / 44 | R: 2 / 14  Neo C: 1 / 28  E: 1 / 28 | SSM: 15 / 117  NSSM: 22 / 41 | Immediate  + Single or Two stage | Implants or TE | 310 / 321 g |
| Yang 2019 | 79 | 47.7 / 55.9 weeks | 90 days | 3 / 2 | - | 4 / 8 | R: 2 / 7  C: 14 / 22 | sacrificing nipple-areolar complex or NSM | Immediate  + Single or Two stage | Implants TE + ADM | Implants  290.94 / 284.07 mL  TE  213.57 / 167.00 mL |
| Talwar 2023 | 86 / 87 | Between January of 2018 and October of 2021 | 9.6 ± 7.1 / 10.1 ± 9.9 mo | 5 / 2 | 38 / 33 | 37 / 39 | Neo R: 4 / 3  R: 26 / 33  Neo C: 42 / 34  C: 33 / 40 | SSM: 114 / 114  NSM: 32 / 32 | Immediate  + Single or Two stage | Implants or TE with ADM  141 / 7 | 541.1 / 525.5 CC |
| Akyurek 2019 | 33 / 22 | Between January of 2009 and December of 2016 | 479 ± 326 / 680 ± 494 days | - | - | 5 / 3 | R: 25 / 20 | - | Immediate  + Two stage | Implants or TE with latissimus dorsi myocutaneous flap | Implants: 482.5 / 470.5 mL  TE: 518.2 / 482.3 mL |
| Manrique 2019 | 100 / 69 | Between January of 2012 and December of 2016 | 17.9 / 17.5 months | 2 / 0 | 4 / 3 | 25 / 19 | R: 32 / 20  C: 70 / 19  E: 81 / 45 | SSM: 63 / 66  NSM: 124 / 58 | Immediate  + Two stage | TE + ADM: 186 / 118 | - |
| Houvenaeghel 2022 | 316 | Between January of 2020 and January of 2022 | - | - | - | 31 / 80 | R: 22 / 56  Neo C: 20 / 39 | SSM: 31 / 137  NSM: 67 / 79 | - | Implants + Mesh or latissimus dorsi myocutaneous flap | - |
| Baker 2018 | 40 | Between March and October of 2016 | 7, 90 days, 3 months after surgery | 0 / 0 | 0 / 0 | 8 / 3 | Neo C: 1 / 0  E: 1 / 2 | NSM: 3 / 2  nipple-sacrificing  : 11 / 13  NSSM: 12 / 4 | Immediate  + Single stage | Implants (silicone)+ ADM | 440 / 295mL |
| Mirhaidari 2020 | 62 | Between February of 2016 and November of 2017 | Between 3 and 24 months / Between 24 and 26 months | 2 / 3 | 9 / 10 | 2 / 7 | - | Sacrificing the nipple-areolar : 71 / 75  NSSM: 41 / 37 | Immediate  + Single stage | Implants + ADM | - |
| Plachinski 2021 | 186 | Between September of  2016 and March of 2019 | 15.59 ± 8.98 / 21.39 ± 12.00 months | 2 / 4 |  | 20 / 31 | R: 17 / 31  Neo C: 24 / 28  C: 13 / 15 | - | Immediate  + Single or Two stage | Implants or TE + ADM | 348.68 / 234.81 mL |
| Bekisz 2022 | 510 | Between March of 2017 through August of 2019 | 20.8 ± 7.4 / 27.5 ± 8.9 months | 6 / 15 | - | 22 / 129 | R: 7 / 26  C: 19 / 91 | SSM: 16 / 86  NSM: 20 / 101  Modified radical : 0 / 2  Total: 40 / 203 | Immediate  + Single or Two stage | Implants + ADM, Mesh or ADM and Mesh | 475 / 416.8 CC |
| ElSherif 2023 | 119 / 201 | Between 2016 and 2019 | 1.45±0.86 / 1.94 ±1.2 years | 2 / 2 | 13 / 24 | 38 / 50 | R: 22 / 44  Neo C: 39 / 64  C: 43 / 98 | NSM | Immediate  + Single or Two stage | Implants + ADM:  201 / 282 | 443 / 435 CC |
| Alcon 2023 | 152 | Between 2014 and 2019 | 7 / 7 months | - | - | - | R: 6 / 22  C: 12 / 43  E：10 / 40 | NSM | Immediate  + Two stage | Implants (silicone,smooth, round) or TE + ADM: | - |
| King 2021 | 228 | Between October of 2014 and January of 2018 | 1.7 ± 0.7 / 2.6 ± 1.2 years | - | - | 57 / 53 | R: 7 / 1  Neo C: 31 / 29  C: 51 / 52 | NSM | Immediate  + Single or Two stage | Implants (silicone or Silicone, smooth, round or Shaped) or TE (smooth or textured) + ADM | 466 / 466.7 CC |
| Braun 2020 | 116 / 44 | Between January of 2015 and June of 2019 | 16 ± 8.3 / 24 ± 13 months | 6 / 0 | 27 / 9 | 3 / 6 | R: 17 / 10  Neo C: 38 / 16  C: 35 / 17  E: 93 / 30 | NSM | Immediate  + Single or Two stage | Implants or TE+ ADM | 280 / 160 CC |
| Avila 2020 | 228 | Between may of  2015 and January of 2018 | - | - | - | 3 / 8 | R: 7 / 1  Neo C: 31 / 29 | NSM | Immediate  + Single or Two stage | Implants or TE+ ADM | - |
| Thangarajah 2019 | 63 | Between 2011 and 2013 | 18 months | 1 / 2 | - | 9 / 5 | R: 3 / 1  Neo C: 8 / 4  C: 7 / 4  E：19 / 14 | SSM: 14 / 15  NSM: 20 / 14 | Immediate | - | 293 / 292 mL |
| Kim 2020 | 167 | Between February of 2015 and February of 2020 | - | 2 / 3 | 3 / 14 | - | R: 6 / 21  Neo C: 3 / 12  C: 17 / 49 | SSM: 6 / 13  NSM: 47 / 104 | Immediate  + Single stage | Implants + ADM | 249.0 / 268.1 CC |
| Klinger 2022 | 67 | Between October of 2018 and September of 2020. | - | - | - | 3 / 0 | Neo C: 2 / 8  C: 4 / 13 | NSSM | Immediate  + Single stage | Implants (Silicone,textured, teardrop-shaped or round) + ADM | - |
| Bettinger 2017 | 110 / 40 | Between June of 2008 and July of 2015 | at 30, 90, 180 days and 6-month after surgery | 14 / 2 | - | 32 / 17 | R: 23 / 8  Neo C: 20 / 4  C: 71 / 25 | SSM: 117 / 42  NSM: 42 / 6  Modified Radical: 6 / 4 | Immediate  + Single stage | Implants + ADM | - |
| Nelson 2022 | 238 | Between December of 2017 and January of 2019 | at 2, 6 weeks and 3-month after surgery | 7 / 7 | 22 / 31 | 33 / 26 | R: 9 / 9  Neo C: 20 / 15  C: 16 / 22 | NSM: 24 / 22 | Immediate  +Single or Two stage | Implants or TE + ADM  96 / 33 | - |
| Kraenzlin 2021 | 286 | Between October of 2016 and June of 2018 | - | 8 / 7 | - | 10 / 3 | Neo R: 12 / 12  R: 20 / 33  Neo C: 23 / 20  C: 37 / 31 | NSM: 95 / 33 | Immediate  + Two stage | TE + ADM  169+77 | - |
| Zhu 2016 | 29 / 59 | Between May of 2012 and October of 2014 | Between 2 and 34 months, with an average of 17.3 months | 0 / 2 | 3 / 13 | 0 / 4 | Neo R: 4 / 16  R: 17 / 25  Neo C: 11 / 14  C: 4 / 10 | SSM: 9 / 71  NSM: 21 / 37 | Immediate  + Two stage | TE + ADM  15 / 50  TE + Inferior dermal flap  3 / 18 | 555 / 587.5 mL |
| Wormer 2019 | 32 / 69 | Between January of 2016 and July of 2017 | 179.3 ± 98.2 / 218.3 ± 119.8 | 0 / 8 |  | 5 / 13 | Neo R: 1 / 1  R: 0 / 11  C: 7 / 14 | NSM: 11 / 13 | Immediate  + Two stage | TE without ADM / TE+ADM | 543.7 / 477.5 mL |
| Walia 2018 | 135 | Between 2011 and 2015 | least 60 days, pain scores at least 30 days | - | - | 2 / 32 | R: 3 / 30  C: 8 / 27 | - | Immediate  + Two stage | TE + ADM | - |
| Escandón 2023 | 154 | Between January of 2011 and December of 2020 | 29.67 / 37.31 months | 6 / 6 | 28 / 29 | - | R: 13 / 14  C: 19 / 19 | SSM: 61 / 62  NSM: 16 / 15 | Immediate  + Two stage | TE (smooth or textured) + ADM | 500 / 400 mL |
| Viezel-Mathieu 2020 | 39 / 38 | Between June 2015 and January 2017 | 163.7 / 634.7 days | 0 / 2 | - | 1 / 2 | R: 12 / 20  Neo C: 12 / 11  C: 11 / 12 | SSM: 19 / 29  NSM: 41 / 27 | Immediate  + Single stage  / Immediate  + Two stage | Implants + ADM / TE + ADM | 397.3 / 415 mL |
| Chandarana 2018 | 61 / 69 | Between January of 2015 and May of 2017 | 8.9 / 19.6 months | - | - | - | R: 19 / 20  C: 32 / 29 | - | Immediate  + Single stage | Implants + ADM | 367 / 290 CC |
| Casella 2014 | 34 / 29 | Between November 2011 and January 2014 | 12 / 13 months | 0 / 0 | - | 0 / 0 | - | SSM: 3 / 5  NSM: 36 / 29 | Immediate  + Single stage | Implants + Mesh | - |
| Bernini 2015 | 34 / 29 | November 2011 and January 2014 | 25 / 26 months | 0 / 0 | - | 0 / 0 | R: 9 / 6 | SSM: 3 / 5  NSM: 36 / 29 | Immediate  + Two stage | TE + Titanium-coated Mesh | - |
| Wow 2020 | 170 | Between March 2019 and October 2021 | 20 months | 0 / 0 | - | 4 / 5 | Neo R: 6 / 0  R: 7 / 17  Neo C: 22 / 46  C: 9 / 10 | SSM: 5 / 42  NSM: 151 / 34 | Immediate  + Single stage  143 / 31  Immediate  + Two stage  13 / 45 | Implants or TE+ Mesh | 375 / 385 mL |
| Sobti 2020 | 20 / 27 | Between January 2015 and May 2018 | 22.9 ± 10.0 / 27.0 ± 11.3 months | - | - | 0 / 0 | Neo R: 4 / 7  R: 16 / 20  Neo C: 14 / 13 | SSM: 8 / 7  NSM: 12 / 20 | Immediate  + Single stage | Implants + Mesh or ADM | 470.0 / 407.4 CC |
| Darrach 2021 | 133 / 89 | Between January of 2017 and April of 2018 | - | - | - | - | - | Bilateral mastectomy: 79 / 45  NSM: 75 / 24 | Immediate  + Two stage | TE+ ADM | - |
| Le 2021 | 64 / 37 | Between 2013 and 2018 | 42 ± 23 months | - | - | 36 / 22 | Neo R: 5 / 3  R: 13 / 5  Neo C: 14 / 9  C: 29 / 17 | - | Immediate  + Single stage | Implants (Smooth or Textured, Silicone or Saline ) + ADM | 350 / 350 CC |
| Cogliandro 2023 | 81 | Between 2013 and 2021 | During the first postoperative month, follow-up consisted of weekly checkups; thereafter, follow-up visits were performed once every 3 months | - | - | - | Neo R: 5 / 3  R: 9 / 14  Neo C: 13 / 13  C: 8 / 20  E: 14 / 38 | NSM | Immediate  + Single stage | Implants (silicone, textured) + Mesh | 467 / 440.1 CC |
| Joon 2021 | 34 | Between February 2018 and September 2019 | preoperatively (T0), and at 2 weeks (T1), 1 month (T2), 3 months (T3), and 6 months post-operation (T4) | - | - | - | R: 5 / 5  C: 6 / 5  E: 19 / 12 | SSM: 5 / 3  NSM: 15 / 11 | Immediate  + Single stage | Implants (Silicone) + ADM | 258.64 / 234.78 CC |

“/” The meaning before and after: is as follows: Prepectoral / Subpectoral

BMI: Body Mass Index

R: Radiotherapy; C: Chemotherapy; H: Endocrinotherapy; Neo: Neoadjuvant

NSM: Nipple-Sparing Mastectomies; SSM: Skin-Sparing Mastectomies; NSSM: Nipple and Skin-Sparing Mastectomies

ADM: Acellular Dermal Matrix; TE: Tissue Expander

CC: Cubic Centimeter; g: Grams; mL: Milliliter
